# Effect of Transparent, Purple, and Yellow Shellac Microcapsules on Properties of the Coating on *Paraberlinia bifoliolata* Surface

**DOI:** 10.3390/polym14163304

**Published:** 2022-08-13

**Authors:** Yan Han, Xiaoxing Yan, Yu Tao

**Affiliations:** 1 Co-Innovation Center of Efficient Processing and Utilization of Forest Resources, Nanjing Forestry University, Nanjing 210037, China; 2 College of Furnishings and Industrial Design, Nanjing Forestry University, Nanjing 210037, China

**Keywords:** shellac, microcapsule, waterborne coating, self-repairing, Beli wood

## Abstract

In order to explore the applicability of the waterborne coating with self-repairing microcapsules based on the surface of wood boards and specify the optimal range of microcapsule content in the coating, three different kinds of shellac microcapsules (transparent shellac, purple shellac, and yellow shellac) were embedded in a waterborne acrylic coating at 0, 1.5 wt.%, 3.0 wt.%, 4.5 wt.%, 6.0 wt.%, and 7.5 wt.%. The Beli wood (*Paraberlinia bifoliolata*) boards were then covered with self-repairing coatings to investigate the self-repairing coating’s physical and chemical properties, aging resistance, and scratch repair abilities. The findings demonstrated that the chromatic difference and gloss of surface coatings on Beli wood boards were significantly influenced by the content of microcapsules. The optical characteristics and cold liquid resistance performance of the coating on Beli wood were enhanced when the microcapsule content was 3.0 wt.%. Additionally, the mechanical qualities of the coating with 3.0 wt.% transparent shellac microcapsules on Beli wood surface were better, with an H hardness, grade 2 adhesion, and 8 kg·cm of impact strength. The studies on scratch repairing and aging resistance indicated that microcapsules helped to slow down the coating’s damage and retard aging. After a microcrack appeared, the waterborne coating with microcapsules on Beli wood’s surface had the capacity to repair itself. After aging, the coating with 3.0 wt.% transparent shellac microcapsule on Beli wood boards had a better performance on the comprehensive properties, with a 28.9% light loss rate and a 6 kg·cm impact resistance. It also had a 25.0% repairing rate in scratch width after being damaged for 5 d. This study advances the development of self-healing waterborne coatings on the wood board with shellac microcapsules by examining the effects of shellac in various colors and shellac microcapsule content in waterborne coatings.

## 1. Introduction

Wood has beautiful patterns [1,2,3,4,5], superior mechanical characteristics [6,7,8], and is environmentally beneficial [9,10]. It is frequently utilized as the main source of everyday goods such as furniture [11,12], architecture [13], and musical instruments [14]. The stability and durability of wood are, however, easily impacted by the external environment because it is a porous natural heterogeneous polymer composite [15]. To protect the substrate, the wood is frequently coated after machining [16,17]. However, due to external forces or the dry shrinkage and wet expansion characteristics of wood [18], the film is susceptible to microcracks. Without human treatment, the microcrack would worsen and become larger, losing the wood surface coating’s protective properties and resulting in unexpected economic losses.

Microcapsule technology is a type of packaging technology that coats gases, liquids, or solids in the form of microscopic particles using natural or synthetic polymer film-forming chemicals [19]. Although there is not a generally applicable coating method for all core materials [20], the microcapsules are still widely used in the fields of medicine [21], food [22], fabrics [23], coatings [24], and more [25] because of the advantages of their structure in completely insulating the core material from the outside environment to preserve the core material’s original performance. With the aid of microcapsules and self-healing technology [26], the self-healing microcapsules are embedded into waterborne coatings that are painted on the surface of the wood in order to effectively inhibit and repair microcracks of waterborne coating and protect the wood board [27]. Xu et al. [28] used phase change materials to load multi-compartment microcapsules synthesized through Pickering emulsion polymerization by encapsulating hydrophobic materials with titanium dioxide nanocapsules. Thermal insulation, antireflection, and self-healing abilities of the coating with microcapsules were all strengths. After adding a polyurea formaldehyde (PU) microcapsule containing cerium nitrate to an epoxy resin coating, Farzi et al. [29] discovered that the epoxy coating would successfully self-repair once it was damaged because the coated cerium nitrate would be released from the microcapsules. To create PU/PANI double-shell microcapsules that are loaded with tung oil, Feng et al. [30] first created tung oil microcapsules enclosed by PU by interfacial polymerization. Then, they deposited polyaniline (PANI) on the surface of the PU microcapsules in situ. It was established that the tung oil core and PANI shell of the microcapsules contributed to the coating’s remarkable self-healing and anti-corrosion capabilities by adding 10 wt.% double-shell microcapsules to the epoxy resin coating. Wu et al. [31] used a one-pot photopolymerization and interfacial aniline polymerization to synthesize the robust PANI microcapsules, which were uniformly dispersed in the waterborne epoxy coating to provide it the self-repairing and anti-corrosion capabilities.

Urea-formaldehyde (UF) resin and melamine-formaldehyde (MF) are widely used as wall materials for preparing microcapsules by in situ polymerization, where the microcapsule walls are formed from the outside of micro core materials (small solid particles or droplets). Compared with UF, MF has superior adhesion, better moisture resistance, and lower formaldehyde emission [32]. The shellac microcapsules encapsulated by MF resin has good self-healing abilities in a waterborne acrylic resin coating [33,34]. Since the 18th century, Beli wood (*Paraberlinia bifoliolata*) has been prized as expensive sawn wood. It benefits from having a high density, good strength, strong resistance to corrosion, and beautiful patterns [35]. It is frequently utilized to create building materials, fine furniture, flooring, and carvings. Under the effect of environmental moisture, it is simple to deform or crack, which results in economic losses. The dimensional instability of wood destroys the coating and causes it to lose its protective role. Therefore, it is crucial to investigate self-healing coatings combined with microcapsule technology in order to retain the stability and usefulness of the coating on wood.

Existing research demonstrates that the shellac microcapsule content has a considerable impact on the physical, chemical, and repair properties of waterborne films [36]. The waterborne film had better all-around qualities when the microcapsule content is less than 6.0 wt.% [37]. The main shellac resin products available on the market are transparent shellac, purple shellac, and yellow shellac. These products vary depending on the various raw materials’ geographic origins and treatment techniques [38]. All the active substances have the same chemical ingredient [39]. However, it has not been made obvious how shellac resin compounds with different colors affect the mechanical, self-repairing, and optical characteristics of waterborne coatings.

Therefore, in this paper, various types of shellac microcapsules (transparent shellac, purple shellac, and yellow shellac) were mixed with the waterborne coating at the contents of 0, 1.5 wt.%, 3.0 wt.%, 4.5 wt.%, 6.0 wt.%, and 7.5 wt.%, and then coated on the surface of Beli wood to investigate the applicability of self-repairing coating of embedded microcapsules on the wood board.

By characterizing and analyzing the optical, mechanical, physical, and chemical properties, aging resistance, and scratch repair performance of prepared waterborne coatings, the types of shellac microcapsules that were most suitable for the self-healing coating on the surface of Beli wood and the optimal content of these microcapsules in the coating were determined. Self-repairing mechanism analysis of the waterborne coating with shellac microcapsules based on the Beli wood surface was illustrated, which contributes to the optimization of shellac microcapsules and the development of self-healing waterborne coatings on the surface of wooden constructions and products.

## 2. Materials and Methods

### 2.1. Test Materials

Both the 37 wt.% formaldehyde solution and ethyl acetate were bought from Xi’an Tianmao Chemical Co., Ltd., Xi’an, China. The emulsifiers Span-20 (sorbitan monolaurate), Tween-20 (polyoxyethylene sorbitan monolaurate), and melamine were bought from Shandong Yousuo Chemical Technology Co., Ltd., Linyi, China. The triethanolamine was purchased from Guangzhou Jiale Chemical Co., Ltd., Guangzhou, China. Rosin was bought from Suzhou Guyue Musical Instrument Co., Ltd., Suzhou, China. Citric acid monohydrate was acquired from Nanjing Quanlong biological hydration Technology Co., Ltd., Nanjing, China. Absolute ethanol was acquired from Wuxi Jingke Chemical Co., Ltd., Wuxi, China. The waterborne coating was purchased from Dulux Coatings Co., Ltd. Shanghai, China. Water, a matting agent, an additive, and waterborne acrylic acid copolymer dispersion were the main components. Transparent shellac, purple shellac, and yellow shellac were among the varieties of 12.5 wt.% pure shellac that were obtained from Shanghai Yuyan Building Materials Co., Ltd., Shanghai, China.

### 2.2. Preparation Method of Microcapsules

Take the microcapsules containing transparent shellac in the core material as an example.

(1) Prepolymerization of the wall material

The 3.5:1 molar ratio of 12.00 g melamine to 27.04 g 37 wt.% formaldehyde solution was combined into a beaker. Then, 60 mL of distilled water was added. The hydroxymethyl melamine mixture was synthesized by constantly stirring the mixture at 600 rpm for 30 min in a 70 °C DF-101s constant temperature water bath (Gongyi Yuhua Instrument Co., Ltd., Zhengzhou, China) after several drops of triethanolamine were applied to adjust the pH of the mixture to 8–9.

(2) Synthesis of the core material

For standby, 8.8 g of each liquid (shellac and rosin) was weighed and equally combined. The beaker was filled with 157.8 mL of absolute ethanol, 0.3 g of Span-20, and 0.3 g of Tween-20. Then, the shellac–rosin mixture was added to the mixture. After thoroughly stirring, the prepared liquid was stirred for 60 min at a speed of 600 rpm in a water bath with a constant temperature of 60 °C to create the core material.

(3) In situ polymerization of microcapsules

Using a stirrer at 600 rpm, the core material emulsion was gradually added to the MF prepolymer solution. The mixture was placed in the BILON-500 ultrasonic material emulsion disperser (Shanghai Bilang Instrument Co., Ltd., Shanghai, China) for 15 min. Following ultrasonic blending, the solution was put into a water bath pot, and the pH of the solution was adjusted with citric acid at a 600 rpm speed. The temperature was gradually raised to 60 °C for a constant temperature reaction for 3 h after the pH level reached approximately 4.5. After 3 d, the resulting mixture was filtered, thoroughly cleaned with deionized water and ethanol using the SHZ-D circulating water multipurpose vacuum pump (Henan Yuhua Instrument Co., Ltd., Zhengzhou, China), and then dried.

The parallel method and amount of shellac solution were used to make microcapsules with purple and yellow shellac.

### 2.3. Preparation Method for Films

Beli (*Paraberlinia bifoliolate*) wood boards (50 mm × 50 mm × 10 mm; length × width × thickness, respectively) were supplied by Beijing Tiantan Furniture Co., Ltd., Beijing, China. The surface of Beli wood boards was sanded along the wood grain direction with 300 mesh (48 μm) sandpaper. Then, a 600 mesh (23 μm) sandpaper was used to fine sand the wood boards in the same way. The sawdust produced by sanding was cleaned with a brush and wiped with a dry cloth for standby.

At the contents of 0, 1.5 wt.%, 3.0 wt.%, 4.5 wt.%, 6.0 wt.%, and 7.5 wt.%, the transparent, purple, and yellow shellac microcapsules were embedded into the waterborne coatings, stirred, and mixed evenly to obtain the self-healing coating (every sample at 4.00 g). The coatings were uniformly applied to the Beli wood board surface using a brush. The samples were dried for 3 h in a cool, well-ventilated environment, and the waterborne film’s surface was sanded using fine sandpaper with a mesh size of 600. The coated boards were placed for 12 h in preparation for further testing after the similar methods were repeated twice.

### 2.4. Testing and Characterization

The gloss was measured using an HG268 gloss meter (Shenzhen Threenh Technology Co., Ltd., Shenzhen, China) in accordance with the Chinese standard GB/T 4893.6-2013 for coatings based on the surface of Beli wood boards [40]. A SEGT-J portable chromatic difference instrument (Zhuhai Tianchuang Instrument Co., Ltd., Zhuhai, China) was employed to examine the coating’s chromaticity value based on the Beli wood surface before and after aging in accordance with Chinese standard GB/T 11186.3-1989. The chromaticity values of the waterborne film without microcapsules were recorded as *L*_1_, *a*_1_, and *b*_1_. The chromaticity values of the waterborne film with microcapsules were recorded as *L*_2_, *a*_2_, and *b*_2_. The ΔE* (chromatic difference) of the waterborne film was determined by averaging the four points from each film. ΔL* indicates lightness difference, Δa* indicates the difference between red and green, and Δb* indicates the difference between yellow and blue. The CIELAB chromatic difference Formula (1) was used to determine the chromatic difference of waterborne coatings containing shellac microcapsules [41,42].

ΔE* = [(ΔL*)^2^ + (Δa*)^2^ + (Δb*)^2^]^1/2^ (1)

A pencil hardness tester was employed to examine the hardness of waterborne film using 6H–6B pencils in accordance with GB/T 6739-2006 [43]. The 6B is the softest, 6H is the hardest, and HB is the middle value. The highest hardness of the pencil was noted as the coating’s hardness before an indentation occurred on the coating’s surface. To evaluate the adhesion grade of the coating, QFH-HG600 film scribing equipment (Shanghai Le’ao Test Instrument Co., Ltd., Shanghai, China) was utilized. There are six grades, of which grade 0 denotes the best adhesion of the coating and grade 5 denotes the worst adhesion of the coating. According to GB/T 1731-1993 [44], the impact resistance of the coating is characterized by a QCJ film impactor tester (Shanghai Le’ao Test Instrument Co., Ltd., Shanghai, China). The JB-4C precise roughness tester (Shanghai Taiming Optical Instrument Co., Ltd., Shanghai, China) was employed to assess the coating’s roughness.

According to GB/T 4893.1-2021 [45], the test solutions for the cold liquid resistance test of coating based on Beli wood surface were soy sauce, iodine, toilet water, and 75% medical ethanol. The coated surface was first cleaned. The test solution was applied to the filter paper. It was removed, and then it was applied to the coating surface in a closed environment. The filter paper was removed after a while, and any remaining liquid on the surface was cleaned. After standing for 18 h, the damage to the coating surface was observed, and the grade was evaluated concerning the grading table (Table 1). By using a chromatic difference meter to record the chromaticity value of the Beli wood surface coating before and after the liquid resistance test, the ΔE* was calculated according to Formula (1).

The chemical composition of microcapsules, wall material, core material, and the waterborne coating on the surface of Beli wood was examined using the VERTEX 80V Fourier transform infrared spectrometer (FTIR, Shanghai Smio Analytical Instrument Co., Ltd., Shanghai, China). The Quanta-200 scanning electron microscope (SEM, FEI Company, Hillsboro, OR, USA) was chosen to characterize the micromorphology of shellac microcapsules encapsulated by MF resin and waterborne coatings on the Beli wood surface. The particle size distribution of microcapsules was measured by the software “Nano measurer”, with a measurement capacity of 100 [46].

To simulate the repair of microcracks on the self-repairing coating based on the surface of Beli wood in daily use, artificial accelerated dry-heat aging tests for the surface coating based on Beli wood were conducted to determine the optimum content of shellac microcapsules in the self-repairing coating suitable for Beli wood. The Beli wood boards coated with self-repairing coatings were placed in a 120 °C DHG-9643BS-Ⅲ drying oven (Shanghai Xinmiao Medical Instrument Co., Ltd., Shanghai, China). The tests were done on the surface coating’s chromaticity and gloss values every 6 h. The ΔE* of the coating based on the Beli wood surface before and after aging was calculated according to the chromatic difference Formula (1), and the light loss rate of the coating before and after aging was calculated according to Formula (2). *G_L_* denotes the light loss rate. *G*_1_ denotes the gloss of waterborne films before aging. *G*_2_ denotes the gloss of waterborne films after aging.

*G_L_* = (*G*_1_ − *G*_2_)/*G*_1_ × 100% (2)

The hardness, adhesion, impact strength, and roughness of the surface coating on Beli wood were examined following the 36 h high-temperature aging test. The procedure was the same as for testing the mechanical properties of Beli wood’s surface coating before aging.

A single-sided blade with a length of 2 cm and a depth of around 100 µm scratched the self-healing coating in a direction parallel to the tensile direction. The healing results at the scratch of the coating were examined using the Zeiss Axio Scope A1 optical microscope (OM, Carl Zeiss AG, Oberkochen, Germany) and SEM both at the time the damage was formed and after 5 d. To assess the self-healing coating’s ability to repair itself, Formula (3) was employed to calculate the change rate of scratch width. *D_H_* denotes the width change rate. *D*_1_ denotes the scratch width of waterborne films before self-repairing. *D*_2_ denotes the scratch width of waterborne films after self-repairing [47].

*D_H_* = (*D*_1_ − *D*_2_)/*D*_1_ × 100% (3)

## 3. Results and Analysis

### 3.1. Microstructure, Particle Size, and Chemical Composition Analysis of Shellac Microcapsules

**Figure 1:**
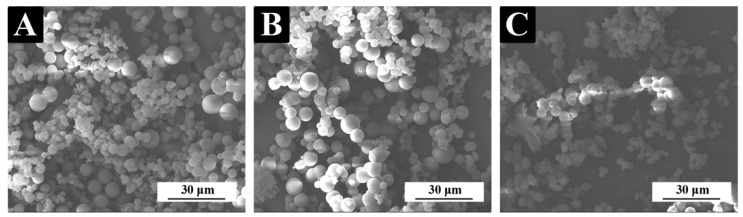
SEM images of (**A**) transparent shellac microcapsules; (**B**) purple shellac microcapsules; (**C**) yellow shellac microcapsules.

**Figure 2:**
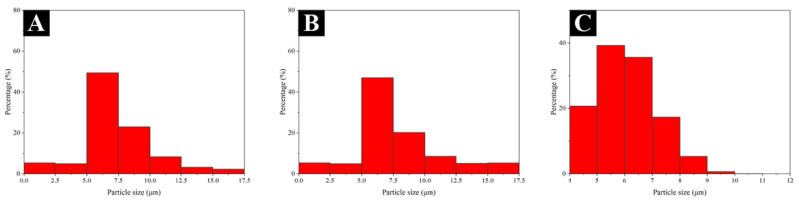
Particle size distribution of (**A**) transparent shellac microcapsules; (**B**) purple shellac microcapsules; (**C**) yellow shellac microcapsules.

**Figure 3:**
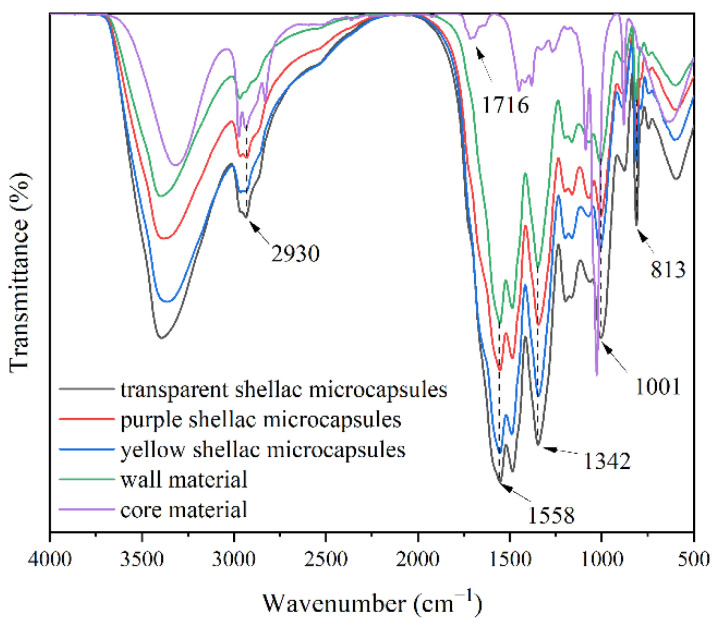
FTIR of the wall material, core material, purple shellac microcapsules, yellow shellac microcapsules, and transparent shellac microcapsules.

Figure 1 displays the micromorphology of three different shellac microcapsules. The microcapsules had smooth surfaces and a spherical appearance, which is consistent with the results in our previous study [48]. The particle size distribution of different shellac microcapsules through the software “Nano measurer” is shown in Figure 2. The transparent shellac microcapsules’ average particle size was 5.7 μm, the purple shellac microcapsules’ average particle size was 7.4 μm, and the yellow shellac microcapsules’ average particle size was 5.3 μm. Figure 3 displays the infrared spectra of the wall material (MF resin), core material (transparent shellac), purple shellac microcapsules, yellow shellac microcapsules, and transparent shellac microcapsules. The characteristic peak of –CH_2_ can be seen on the core curve at 2930 cm^−1^, and the telescopic vibration of C=O can be seen on the peak at 1716 cm^−1^, which indicates that the microcapsule curves have shellac’s characteristic peaks [49]. The stretching vibration absorption peak was at 1558 cm^−1^ which is probably due to melamine ring distortion [50], the stretching vibration absorption peak of C–N in the aromatic ring was at 1342 cm^−1^, the absorption peak formed by the plane bending vibration of C–H in the aromatic ring was at 1001 cm^−1^, and the bending vibration absorption peak of the triazine ring was at 813 cm^−1^. Therefore, the transparent shellac, purple shellac, and yellow shellac microcapsules coated with MF resin were successfully synthesized.

### 3.2. Effect of Shellac Microcapsules on the Chromatic Difference and Gloss of the Coating on the Beli Wood Surface

The chromaticity value of the coating with microcapsules on the Beli wood surface was compared with that of the coating without microcapsules on the Beli wood surface. The variation in the ΔE* of coatings with the different content of different shellac microcapsules based on the Beli wood surface is shown in Table 2. The chromatic difference of the coating based on the Beli wood surface generally rose with an increase in microcapsule content. The prepared microcapsules were light yellow because of the color of the shellac used as the core material. Therefore, the coating’s appearance color and ΔE* value were more dramatically impacted by the number of microcapsules. Moreover, the chromatic difference of the coating based on the Beli wood surface was significantly influenced by the type of microcapsules in the coating. The color of the microcapsule powder can be influenced by the color of the shellac used as the core material, which can alter the color of the coating. The coating based on the Beli wood surface embedded with purple shellac microcapsule had the smallest change in ΔE* of all of them. The minimum ΔE* of coating based on the Beli wood surface with transparent shellac microcapsules was 8.33 when the microcapsule content was 3.0 wt.%. The increase in the ΔE* of the coating based on Beli wood became greater and quicker when the content of microcapsules exceeded 3.0 wt.%.

Table 3 shows the significance analysis of ΔE* of these samples. In Table 3, SS stands for the sum of squares from the mean square, reflecting the sum of squares between groups (microcapsule content or microcapsule type) and within groups. d_f_ is for the degree of freedom, and MS is the mean square that is produced by dividing the sum of squares by the degrees of freedom. The test statistic, or F, is what is utilized to calculate the hypothesis test. The *p*-value, which denotes the degree of significance, is used to assess the range and interval of overall parameters and determine the likelihood that an experiment will actually take place. The F value at the appropriate level of significance is represented by F_crit_. There is no difference between the two groups of data when F is less than F_crit_. The difference is highly significant if *p*-value ≤ 0.01. If 0.01 < *p*-value < 0.05, then the difference is considered significant according to the standard criteria. There is no difference if *p*-value is greater than 0.05. For the microcapsule content, F > F_crit_ and 0.01 < *p*-value < 0.05, meaning that the difference among the ΔE* of coatings with different microcapsule contents was significant. For the microcapsule type, F < F_crit_ and *p*-value > 0.05, meaning there was no difference among the date of different microcapsule types.

Table 4 shows the variation in the gloss of coating based on the Beli wood surface with the content of different shellac microcapsules in the coating. The existence of microcapsules caused the decline in the gloss of the waterborne coating on Beli wood under 20°, 60°, and 85° incident light. The gloss of the coating based on the Beli wood surface was reduced as the microcapsule content in the coating rose. This is because an increase in microcapsule particles enhances the coating’s diffuse reflection of light. Among them, when the microcapsule content was 1.5–3.0 wt.%, the gloss of the coating decreased slowly. When the content exceeded 3.0 wt.%, the gloss of the waterborne coating based on the Beli wood surface decreased greatly. Under the three different incident angles, the gloss of the waterborne coating was the highest at 60° incident light. At 60° incident light, the gloss of the transparent shellac microcapsule coating was least impacted by the microcapsule content. The coating embedded with transparent shellac microcapsules had the highest gloss out of the three types of shellac microcapsules. The gloss of coating with transparent shellac microcapsules reached 47.0% when the content was 3.0 wt.%. Table 5 shows the significance analysis of the gloss of different samples. For the microcapsule content, F > F_crit_ and *p*-value = 0.01, meaning that the difference among the gloss of coatings with different microcapsule contents was highly significant. For the microcapsule type, F < F_crit_ and *p*-value > 0.05, meaning there was no difference among the date of different microcapsule types.

Therefore, the content of microcapsules in coatings had a significant impact on ΔE* and gloss, which is consistent with previous research [33,51], while the color of the microcapsule with different core materials had less influence. The optical properties of the waterborne coating on Beli wood were better when the content of the microcapsule was 3.0 wt.%.

### 3.3. Effect of Shellac Microcapsules on Mechanical Properties of the Coating Based on the Beli Wood Surface

Table 6 indicates the change in hardness of the waterborne coating on Beli wood with the content of different shellac microcapsules embedded in the coating. The hardness of the waterborne coating on Beli wood was generally soft. The coating’s hardness was B in the absence of microcapsules. It was discovered through comparison and observation of coatings with various microcapsule contents that the hardness of coatings based on the Beli wood surface gradually rose with a relatively small change range. When the number of transparent shellac microcapsules rose from 0 to 7.5 wt.%, the coating hardness only went up from B to 2H. This might be the case since the coating’s material characteristics control the coating’s hardness. As fillers, the microcapsules have little impact on coating hardness; hence, there was little change in coating hardness. The addition of microcapsules made of various shellac materials had a similar effect on the coating’s hardness when the number of microcapsules in the coating was fixed.

The change in adhesion of the coating on Beli wood with the content of different shellac core microcapsules in the coating is shown in Table 7. The type of microcapsules in the coating has a minimal bearing on how well the coating adhered when the microcapsule concentration remained constant. However, when the microcapsule types were the same, the coating’s adhesion was reduced as the microcapsule number rose. When the microcapsule content was 6.0 wt.%, the adhesion of the coating significantly decreased, from grade 1 to grade 3 for the transparent shellac microcapsule coating, grade 1 to grade 2 for the purple shellac microcapsule coating, and grade 1 to grade 4 for the yellow shellac microcapsule coating. This might be due to the various kinds and concentrations of impurities found in shellac products with various colors, which are brought on by different decolorization processes and degrees [38]. When the waterborne acrylic coating and the addition of microcapsules remain the same, the dense structure inside the coating is broken, which lowers the coating’s cohesiveness and the adhesion between the coating and the wood board. Therefore, a waterborne coating’s effectiveness in terms of adhesion is impacted by an excessive microcapsule content. When the number of microcapsules was 1.5–3.0 wt.%, according to Table 5, the coating adhered better.

The impact resistance of coating with the content of different shellac microcapsules based on the Beli wood surface is shown in Table 8. The type and content of the microcapsules had an impact resistance that differed as well. The impact strength of waterborne coatings with transparent shellac microcapsule and yellow shellac microcapsule first increased and then declined as microcapsule content was increased. When the microcapsule content was 3.0 wt.%, the maximum impact strength of these two microcapsule coatings was 8 kg·cm. The impact strength of the waterborne coating containing purple shellac microcapsules remained consistent at first and then gradually dropped as the number of microcapsules in the coating increased. When there were too many microcapsules in the coating, the microcapsules were unevenly distributed, which made the stress concentration easily occur in the coating, and the coating was more likely to break when it was impacted, which was reflected in the reduction of the impact resistance. Therefore, the impact resistance of the coating was better when 3.0 wt.% transparent shellac microcapsule or yellow shellac microcapsule was added.

**Figure 4:**
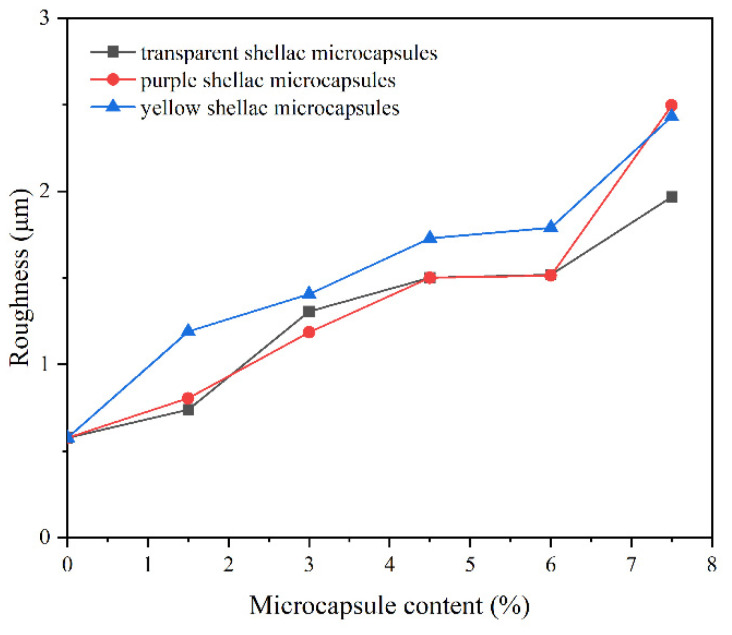
The roughness of the coating on Beli wood with the content of different shellac core microcapsules.

The roughness of the surface coating based on Beli wood changed with the content of different shellac microcapsules in the coating, as shown in Figure 4. It is obvious from the figure that as the number of microcapsules increased, so did the roughness of the surface coating on the wood board. The prepared microcapsules were spherical particles. They were mixed with waterborne coating and coated on the surface of Beli wood, resulting in some gaps among microcapsules. The microcapsules became denser as the microcapsule content rose, and the number of gaps increased, resulting in increased inhomogeneity and roughness of the whole coating. The waterborne coating with transparent shellac microcapsules had less roughness despite having the same microcapsule concentration. When the transparent microcapsule content was 1.5 wt.%, the roughness of the coating was 0.74 μm. It was a little rougher than the coating without microcapsules, which had a roughness of 0.58 μm. This might be because the transparent shellac microcapsules have a smaller particle size. The waterborne coating with 3.0 wt.% transparent shellac microcapsules exhibited improved mechanical properties on the surface of the Beli wood board.

### 3.4. Effect of Microcapsules Coating Different Shellac on Cold Liquid Resistance of Coating Based on the Beli Wood Surface

One of the key indicators of a film’s physical and chemical characteristics on the surface of wooden boards is the waterborne film’s resistance to liquids. Before and after the cold liquid resistance test, the ΔE* of the surface coating on Beli wood with the content of different shellac microcapsules in the coating is shown in Table 9. After the liquid resistance test, the coating with microcapsules had a smaller ΔE* than the coating without microcapsules. When the content of microcapsules in the coating was 1.5 wt.%, the liquid resistance ΔE* after testing by soy sauce, toilet water, iodine, and 75% medical ethanol was smaller.

The liquid resistance results of the surface coating on Beli wood are shown in Table 10. For the coating with 0–7.5 wt.% different shellac microcapsules, the liquid resistance grade for toilet water and 75% medical ethanol was grade 1. However, most of the cold liquid resistance grades for soy sauce and iodine were grade 2, and a few were grade 3. On the coating’s surface, there was a slight discoloration. It was primarily because iodine is dark brown and soy sauce is reddish-brown in color, making it easy to dye the coating. As the number of microcapsules rose, the coatings also had larger surfaces, which improved the interaction between the self-healing coating and the dye and made it simpler for the liquid color to penetrate the coating. According to Table 8, the waterborne coating had the best liquid resistance when the microcapsules content in the coating was 0–3.0 wt.%.

### 3.5. Effect of Shellac Microcapsules on the Micromorphology of the Coating on the Beli Wood Surface

**Figure 5:**
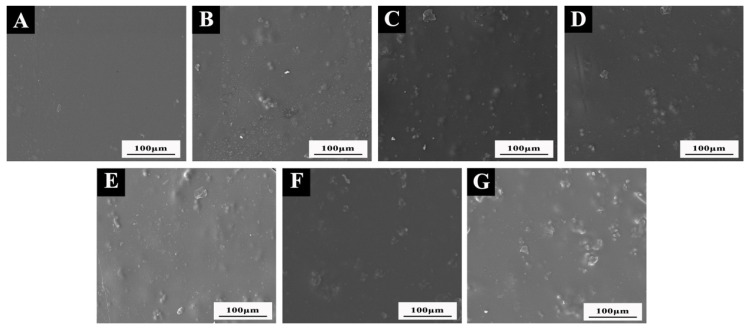
SEM of coatings with (**A**) 0 microcapsules; (**B**) 3.0 wt.% transparent shellac microcapsules; (**C**) 6.0 wt.% transparent shellac microcapsules; (**D**) 3.0 wt.% purple shellac microcapsules; (**E**) 6.0 wt.% purple shellac microcapsules; (**F**) 3.0 wt.% yellow shellac microcapsules; (**G**) 6.0 wt.% yellow shellac microcapsules.

Combined with the above test results, the microcapsule contents of 3.0 wt.% and 6.0 wt.% were selected because of their better comprehensive performances, and the micromorphology of the coating with microcapsules on the surface of Beli wood was analyzed. The results are shown in Figure 5. The coating had a smooth, flat surface. In Figure 5B–G, microcapsule particles could be seen on the coated surface. When the content of microcapsules increased, the particles agglomerated in the coating, the dispersion decreased, the microcapsule powder on the coating surface bulged obviously, and the surface morphology of the coating with 6.0 wt.% microcapsules was worse than that of the coating with 3.0 wt.% microcapsules. When the content was 3.0 wt.%, the dispersion of transparent shellac microcapsules in the coating was the best and the microcapsule powder was evenly distributed, while the yellow shellac microcapsules agglomerated obviously in the coating and had poor dispersion. Therefore, the micromorphology of the coating with 3.0 wt.% transparent shellac microcapsules on the surface of Beli wood was relatively good.

### 3.6. Effect of Shellac Microcapsule on Aging Repair Performance of the Coating Based on the Beli Wood Surface

#### 3.6.1. Analysis of Microstructure and Chemical Composition before and after Aging

**Figure 6:**
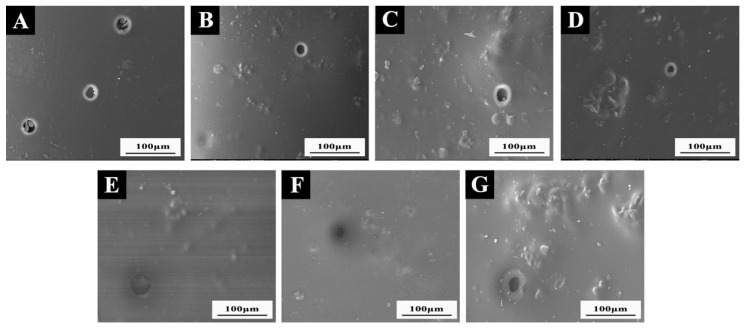
SEM of coatings with different shellac microcapsules after aging: (**A**) 0 microcapsules; (**B**) 3.0 wt.% transparent shellac microcapsules; (**C**) 6.0 wt.% transparent shellac microcapsules; (**D**) 3.0 wt.% transparent purple microcapsules; (**E**) 6.0 wt.% purple shellac microcapsules; (**F**) 3.0 wt.% yellow shellac microcapsules; (**G**) 6.0 wt.% yellow shellac microcapsules.

Figure 6 displays the SEM image of the surface coating on Beli wood following the high-temperature aging test at 120 °C. Compared with the SEM image before aging (Figure 5), it can be observed that after high-temperature aging, the coating surface was damaged to a certain extent, resulting in bubbles and cracks. The pure waterborne coating without microcapsules (Figure 6A) had more damage and a larger crack diameter after aging at 120 °C. After high-temperature aging, the coating with shellac microcapsules on Beli wood also produced a small number of round hole cracks, but the damaged area was small. After microcracks occurred in the coating under the action of a high-temperature environment, the shellac microcapsules around the cracks were damaged by force. After the repair agent flowed out, it could inhibit the continuous expansion of microcracks and repair the cracks to a certain extent. The environmental resistance of the waterborne coating was improved, while the waterborne coating without microcapsules had poor environmental tolerance, resulting in the coating being seriously damaged. After high-temperature aging, the aging degree of the coating with different shellac microcapsules was the same. Therefore, adding self-healing shellac microcapsules contributed to improving the environmental tolerance of the coating on Beli wood surface and the repairability of microcracks.

**Figure 7:**
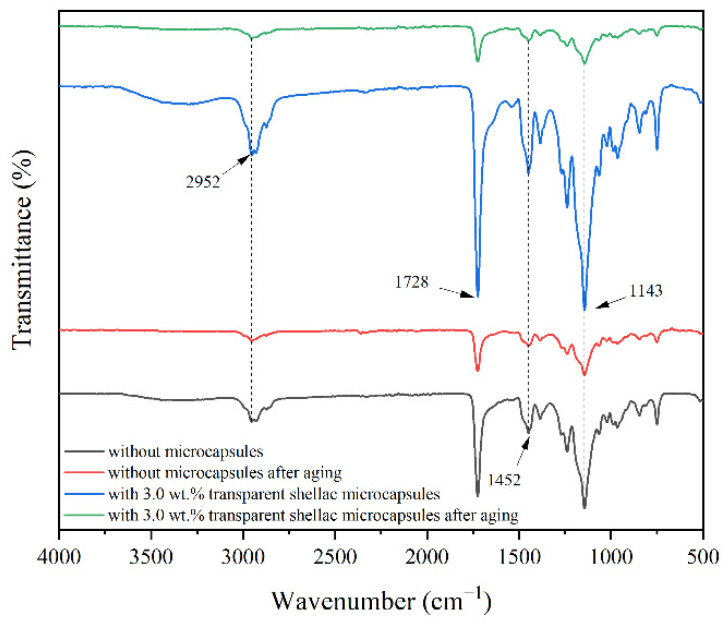
FTIR of the coatings with and without transparent shellac microcapsule before and after aging.

**Figure 8:**
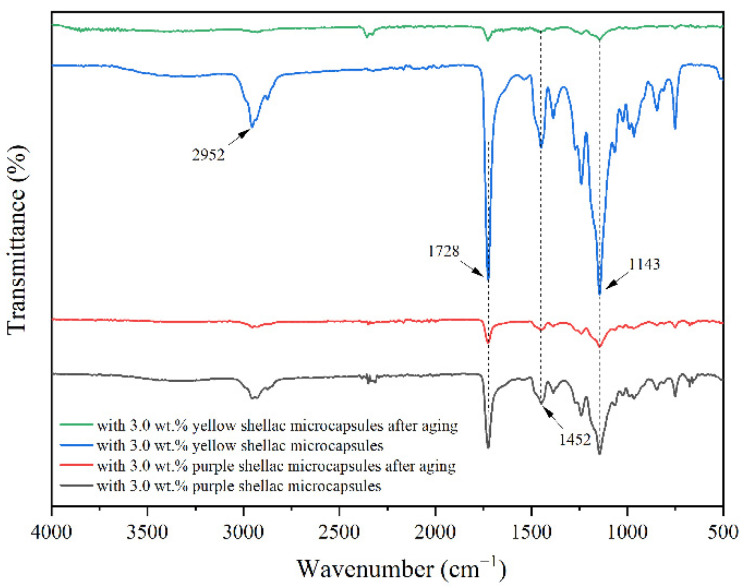
FTIR of the coatings with purple and yellow shellac microcapsule before and after aging.

Figure 7 indicates the infrared spectrum of surface coatings on Beli wood without microcapsules and with 3.0 wt.% transparent shellac microcapsules before and after aging. The infrared spectra of surface coatings on Beli wood with 3.0 wt.% purple shellac microcapsules and yellow shellac microcapsules before and after aging are shown in Figure 8. At 2952 cm^−1^ and 1452 cm^−1^, there were the characteristic peaks of C–CH_3_ in C–H. At 1728 cm^−1^, it was the characteristic peak of the C=O group in the waterborne acrylic resin coating. At 1143 cm^−1^, it was the characteristic absorption peak of C–O in the ester group. According to Figure 7 and Figure 8, it can be found that the infrared spectrum curve trends of the waterborne coating without microcapsules and with microcapsules were the same. The existence of microcapsules in the waterborne coating did not change the chemical properties of the waterborne coating.

According to Figure 7 and Figure 8, it is found that the infrared curves of the coating before and after aging were the same, only the peak intensity was enhanced and weakened, and there was no peak appearance and disappearance. This indicated that the chemical composition of the waterborne coatings without microcapsules and with different shellac microcapsules on the surface of Beli wood had no change before and after high-temperature aging.

#### 3.6.2. Analysis of ΔE* and Gloss Change before and after Aging

**Figure 9:**
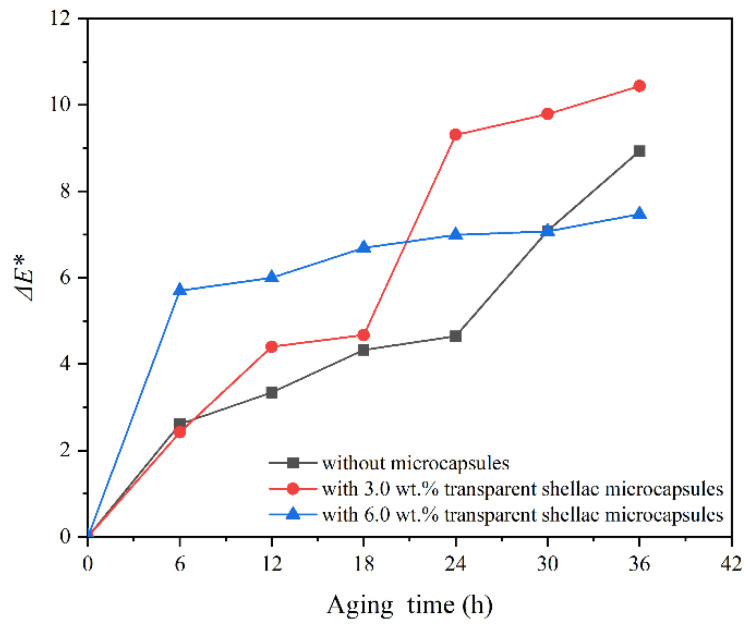
The ΔE* of the coating on Beli wood with transparent shellac microcapsules after aging.

**Figure 10:**
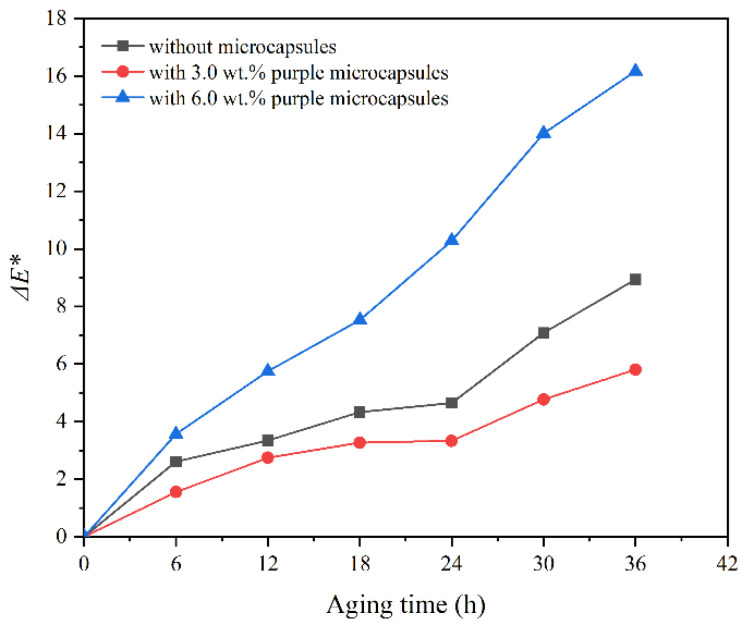
The ΔE* of the coating on Beli wood with purple shellac microcapsules after aging.

**Figure 11:**
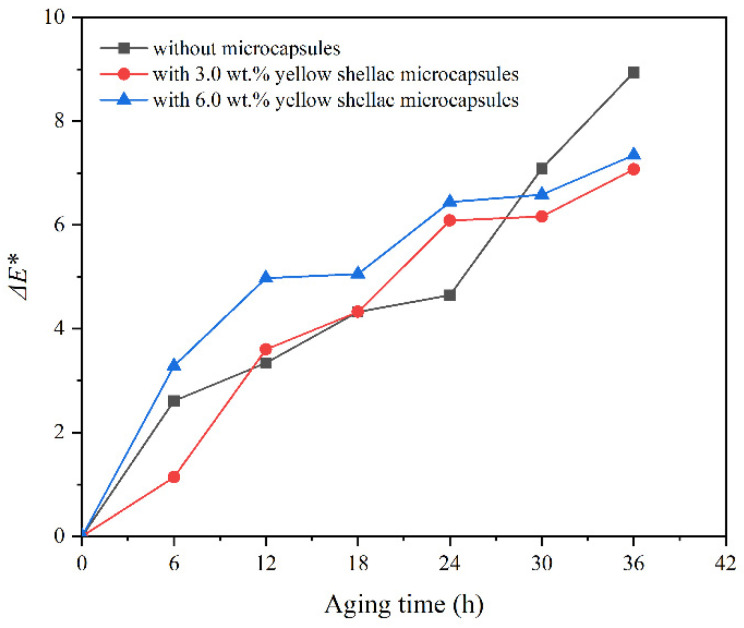
The ΔE* of the coating on Beli wood with yellow shellac microcapsules after aging.

After high-temperature aging, the change in ΔE* of the coating with transparent shellac microcapsules on the surface of Beli wood with aging time is shown in Figure 9. The change in ΔE* of the coating with purple shellac microcapsules with aging time is shown in Figure 10. The ΔE* change of the coating with yellow shellac microcapsules with aging time is shown in Figure 11. Under the high-temperature environment of 120 °C, with the prolonged aging time, the ΔE* value of coatings based on the Beli wood surface was increased, which was due to the long-term high-temperature heat treatment accelerating the aging of Beli wood and surface coating. According to Figure 9, the ΔE* of the waterborne coating with 6.0 wt.% transparent shellac microcapsule after aging for 36 h was less than that of the pure waterborne coating. From Figure 10 and Figure 11, the ΔE* change of the waterborne coating without microcapsules was greater than that of the waterborne coating containing 3.0 wt.% shellac microcapsules. The ΔE* change of the waterborne coating with 3.0 wt.% shellac microcapsules was less than that of the coating with 6.0 wt.% shellac microcapsules. The ΔE* of a pure waterborne coating on the surface of Beli wood reached 8.9 after 36 h of high-temperature heat treatment, compared to 7.5 for a coating with 6.0 wt.% transparent shellac microcapsules, 5.8 for a coating with 3.0 wt.% purple shellac microcapsules, and 7.1 for a coating with 3.0 wt.% yellow shellac microcapsules. Therefore, adding microcapsules to the waterborne coating on the surface of Beli wood could slow down the aging progress of the coating and make the ΔE* value of the coating more stable. When the number of microcapsules was 3.0 wt.%, the overall effect was at its greatest. The change in ΔE* before and after coating aging was also somewhat influenced by the color of the microcapsule’s core material.

**Figure 12:**
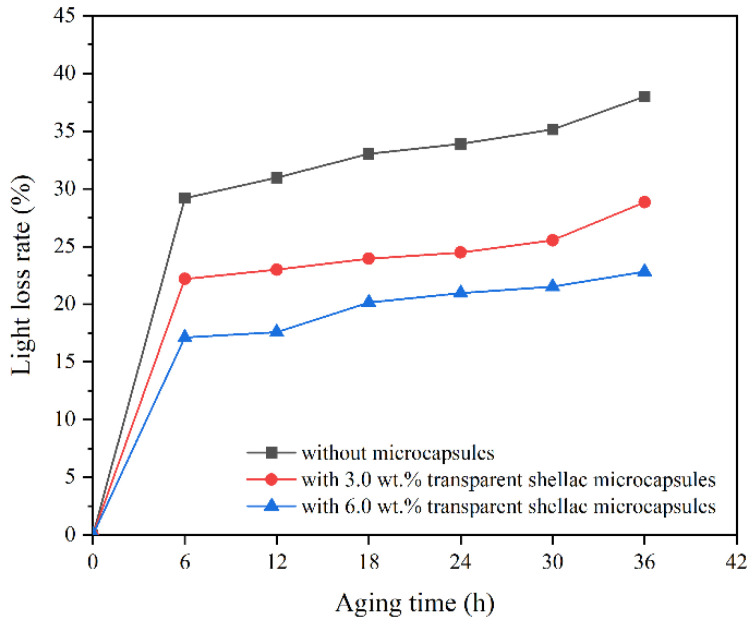
The light loss rate of the coating on Beli wood with transparent shellac microcapsules after aging.

**Figure 13:**
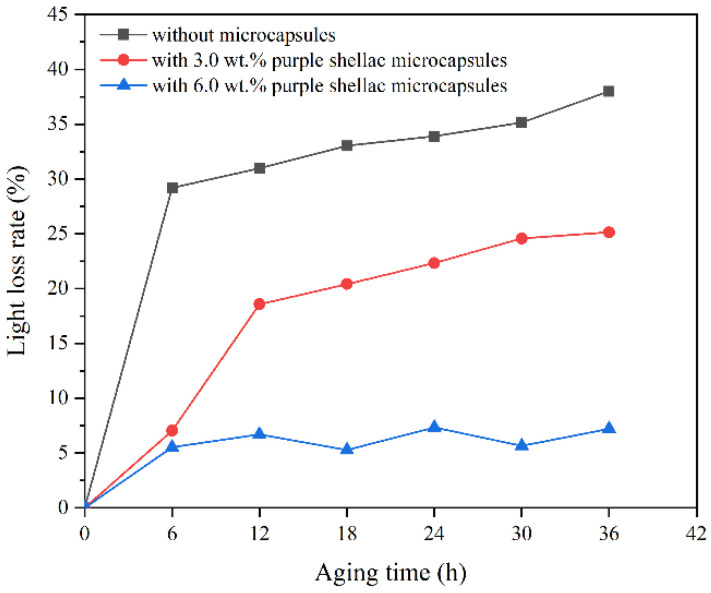
The light loss rate of the coating on Beli wood with purple shellac microcapsules after aging.

**Figure 14:**
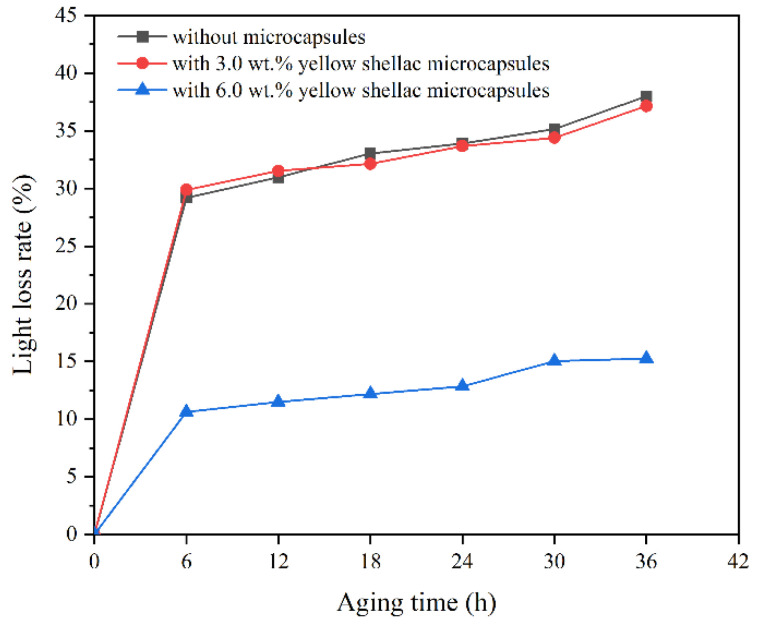
The light loss rate of the coating on Beli wood with yellow shellac microcapsules after aging.

The gloss of the coating is an important index to evaluate its ability to reflect sunlight. The calculation results of the light loss rate of the coating with different microcapsules after aging at 60° incident light are shown in Figure 12, Figure 13 and Figure 14. With the prolonged aging time, the gloss of the coatings on the Beli wood surface decreased, and the light loss rate of the coatings on Beli wood surface was increased. When treated at a high temperature for 6 h, the light loss rate of the waterborne coating on Beli wood was the fastest. After 36 h of high-temperature heat treatment, the light loss rate of pure waterborne coating on Beli wood reached 38.0%, the light loss rate of the waterborne coating on Beli wood with 3.0 wt.% transparent shellac microcapsules was 28.9%, the light loss rate of the waterborne coating on Beli wood with 3.0 wt.% purple shellac microcapsules was 25.1%, and the light loss rate of the waterborne coating on Beli wood with 3.0 wt.% yellow shellac microcapsules was 37.1%. The light loss rate of the pure waterborne coating on the surface of Beli wood was significantly higher than that of the coating with different shellac microcapsules. This might be because the coating with microcapsules was damaged during high-temperature aging, and the shellac interacted with the environment to solidify to repair cracks, which reduced the light loss rate of the waterborne coating on Beli wood.

The research results of the gloss before and after aging were consistent with the results of ΔE* changes. Shellac microcapsules contributed to alleviating the damage and aging of the waterborne coating, slowing down the speed of ΔE* and gloss change, and prolonging the service life of the coating.

#### 3.6.3. Analysis of Mechanical Results before and after Aging

The mechanical qualities of the coating on Beli wood before and after aging in a 120 °C drying oven for 36 h are displayed in Table 11. After high-temperature aging, the hardness of the pure waterborne coating on Beli wood and the waterborne coating with shellac microcapsules was improved, and the adhesion showed a downward trend or remained unchanged. After aging, the impact strength of pure waterborne coating decreased significantly from 7 kg·cm to 4 kg·cm. At the same time, the roughness increased significantly from 0.58 μm to 0.76 μm. However, the impact strength of coating with shellac microcapsules did not decrease significantly. When 3.0 wt.% transparent shellac microcapsules or 3.0 wt.% purple shellac microcapsules were added, the roughness decreased. The shellac microcapsules could effectively alleviate the decline of mechanical qualities of the coating after aging, improve the aging resistance, and maintain the original mechanical strength.

### 3.7. The Repair Performance of Microcapsules and the Interaction between Microcapsules and Wood

#### 3.7.1. Repair Performance of Microcapsules in the Coating on the Beli Wood Surface

**Figure 15:**
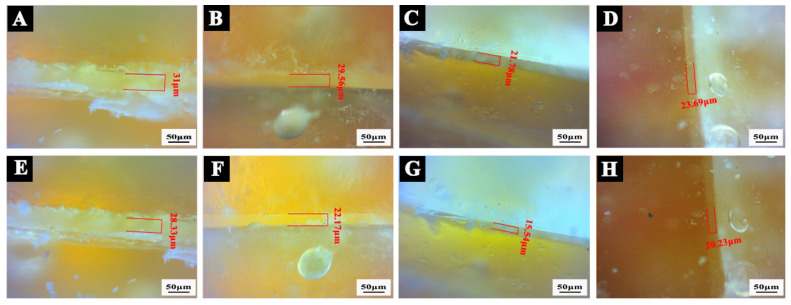
OM of scratch repair results of surface coatings on Beli wood loaded with 3.0 wt.% different shellac microcapsules: (**A**) damaged pure waterborne coating, (**B**) damaged waterborne coating containing transparent shellac microcapsules, (**C**) damaged waterborne coating containing purple shellac microcapsules, (**D**) damaged waterborne coating containing yellow shellac microcapsules, (**E**) damaged pure waterborne coating after 5 d, (**F**) damaged waterborne coating containing transparent shellac microcapsules after 5 d, (**G**) damaged waterborne coating containing purple shellac microcapsules after 5 d, (**H**) damaged waterborne coating containing yellow shellac microcapsules after 5 d.

**Figure 16:**
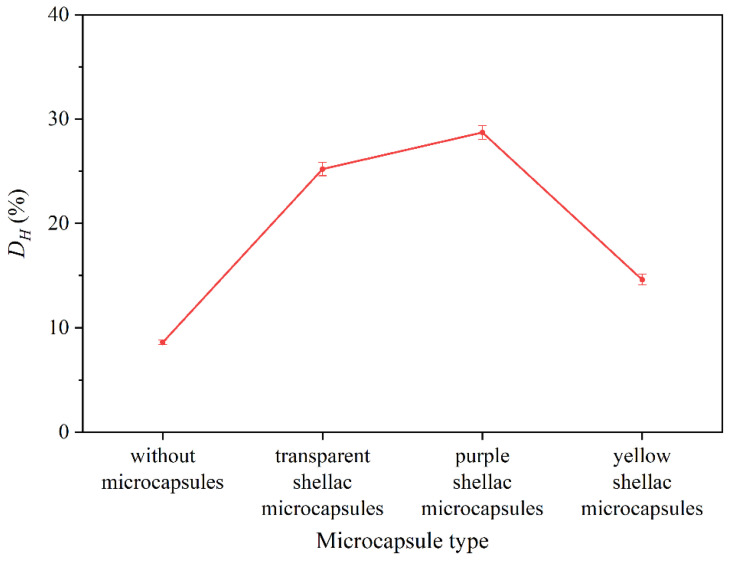
Results of width change rate before and after repair.

To verify the applicability of microcapsules in the coating on the wood board and explore the response relationship between the repair performance of microcapsules in the coating and wood, the scratch repair tests were carried out by artificially damaging self-healing waterborne coating with different shellac microcapsules on Beli wood. The scratch repair results of surface coatings on Beli wood with different shellac microcapsules are shown in Figure 15. The change rate of crack width is shown in Figure 16. According to Figure 15 and Figure 16, after the waterborne coating without microcapsules was scratched by an external force, it was left for 5 d, and the cracks did not change, which indicated that it could not be repaired by itself. After 5 d, the scratch width of the waterborne coating with transparent shellac microcapsules based on Beli wood surface was reduced from 29.56 μm to 22.17 μm, with a width change rate of 25.0%. The scratch width of the waterborne coating with purple shellac microcapsules based on Beli wood surface reduced from 21.78 μm to 15.54 μm, with a width change rate of 28.7%. The scratch width of the waterborne coating with yellow shellac microcapsules based on Beli wood surface was reduced from 23.69 μm to 20.23 μm, with a width change rate of 14.6%. According to our previous research [48], for self-repairing waterborne coatings themselves which were not coated on the wood boards, after 5 days, the waterborne coating with transparent shellac microcapsules had a change rate of scratch width of 39.7%, which with purple shellac microcapsules was 29.3%, and which with yellow shellac microcapsules was 73.0%. The coating on the surface of Beli wood performed less of the self-repairing ability, which indicates that the effect of self-repairing microcapsules in the surface coating is somewhat influenced by the wood board.

Therefore, embedding microcapsules into waterborne coatings contributed to achieving self-repairing when cracks occurred and avoiding further expansion of cracks and more serious damage to the waterborne coating. The repair effect of microcapsules in the coating was affected differently by different colors of shellac materials. Among all samples, the repair effect of coatings with purple and transparent shellac microcapsules was better, followed by yellow shellac microcapsules.

#### 3.7.2. Self-Repairing Mechanism Analysis of the Coating Based on the Beli Wood Surface

**Figure 17:**
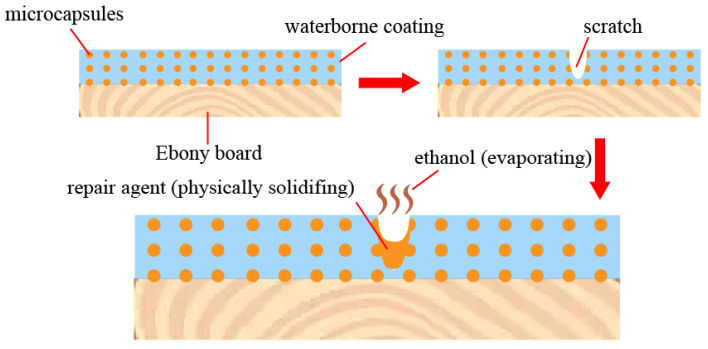
Repair mechanism diagram of microcapsule self-repairing coating on Beli wood.

**Figure 18:**
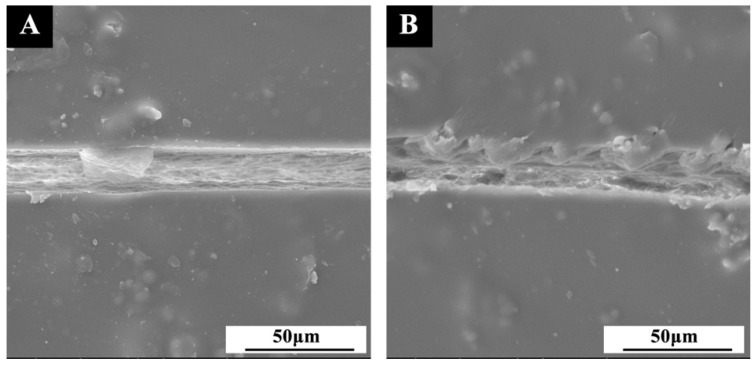
SEM of self-repairing coating with 3.0 wt.% transparent shellac microcapsules: (**A**) when cracks occurred; (**B**) after 5 d.

The repair mechanism of the coating based on the Beli wood surface is shown in Figure 17. After the artificial scratch was formed on the surface coating, the microcapsules embedded in the coating were damaged by force, and the core material (shellac, rosin, and ethanol) was released. The core material repair agent was exposed to the air. With the solvent (ethanol) volatilizing, the core material solidified into a film, which contributed to isolating the board surface from the external environment, repairing the crack, and preventing the further expansion of the crack. Before and after repairing for 5 d, the SEM images of the cracks are shown in Figure 18. It is obvious that when the crack occurred, the section of the coating crack was flat. After 5 d, the section expanded inward, and there were chemicals that filled the gap. This is the process of core material shellac physical curing and filling microcracks, which achieves the repair effect.

## 4. Conclusions

In this paper, the physicochemical properties and self-repairing performance of the coatings with different shellac microcapsules on Beli wood were studied. The test results of physical and chemical properties showed that the content of microcapsules significantly influenced the chromatic difference and gloss of coating based on the Beli wood surface, and the color of shellac had less of an impact. When the content of microcapsules was 3.0 wt.%, the optical properties of the waterborne coating on Beli wood were better. The waterborne coating with transparent shellac microcapsules on Beli wood had better comprehensive mechanical properties and cold liquid resistance, with the hardness of H, adhesion of grade 2, and impact strength of 8 kg·cm. The shellac microcapsules in the surface coating on Beli wood contributed to alleviating the damage and aging of the coating and slowing down the decline in the physical and chemical properties. The light loss rate of 3.0 wt.% transparent shellac microcapsule coating was 28.9%, and the impact resistance of the same sample was 6 kg·cm. Based on the above conclusions, the coating with 3.0 wt.% transparent shellac microcapsules on the surface of Beli wood had good physical properties, chemical properties, and self-healing properties. The change rate of scratch width of the waterborne coating with transparent shellac microcapsules on the surface of Beli wood was 25.0%. The self-healing coating with microcapsules coated on Beli wood had a self-healing ability after cracks occurred, and its self-healing ability was less than the waterborne coating with transparent shellac microcapsules itself, meaning the wood boards as substrates have a certain impact on the effect of the self-healing microcapsules embedded in surface coatings. The causes of the different microcapsule performances brought on by the shellac’s various colors are not easy to accurately determine. Future research will try to clarify it to offer an additional theoretical explanation for the use of the shellac repairing agent in self-repairing coatings.

## Figures and Tables

**Table 1:** Classification of liquid resistance of the paint film on the wood surface.

Grade	Appearance of Paint Film
1	The test area cannot be distinguished from adjacent areas.
2	The test area can be distinguished from adjacent areas only when the light source is projected onto the test surface and reflected into the observer’s eyes.
3	From several directions, the test area can be distinguished from adjacent areas.
4	From all visible directions, the test area can be clearly distinguished from adjacent areas.
5	The test surface structure changed significantly.

**Table 2:** The ΔE* of coatings with the different content of different shellac microcapsules based on the Beli wood surface.

Microcapsule Type	Microcapsule Content (%)	ΔL*	Δa*	Δb*	ΔE*
Transparent Shellac Microcapsules	1.5	7.30	−3.38	−2.23	8.35
	3.0	7.15	−0.10	−4.275	8.33
	4.5	5.60	−4.54	−11.80	13.83
	6.0	11.00	1.53	−2.75	11.44
	7.5	12.40	−3.18	−8.43	15.33
Purple Shellac Microcapsules	1.5	0.25	0.10	−7.45	7.45
	3.0	1.17	−3.54	−7.13	8.05
	4.5	5.40	−2.51	−6.57	8.86
	6.0	8.75	−0.38	2.53	9.10
	7.5	9.97	−3.84	0.77	10.71
Yellow Shellac Microcapsules	1.5	2.30	−1.61	−1.20	3.05
	3.0	2.13	−0.35	−4.33	4.83
	4.5	1.96	−0.38	−6.43	6.73
	6.0	9.37	−3.68	−9.03	13.52
	7.5	4.63	−5.74	−13.2	15.12

**Table 3:** Significance analysis of ΔE* of coatings with the different content of different shellac microcapsules based on the Beli wood surface.

Different Source	SS	d_f_	MS	F	*p*-value	F_crit_
Microcapsule Content	112.44	4.00	28.11	4.59	0.03	3.84
Microcapsule Type	24.64	2.00	12.32	2.01	0.20	4.46
Error	48.96	8.00	6.12	-	-	-
Total	186.04	14.00	-	-	-	-

**Table 4:** The gloss of coating on Beli wood with the content of different shellac microcapsules in the coating.

Microcapsule Type	Microcapsule Content (%)	Gloss (%)		
		20°	60°	85°
Transparent Shellac Microcapsules	0	12.2	58.0	64.4
	1.5	11.3	37.4	37.5
	3.0	11.7	47.0	44.3
	4.5	3.3	20.6	17.9
	6.0	5.1	21.5	18
	7.5	3.8	20.5	15.2
Purple Shellac Microcapsules	1.5	9.6	36.5	35.2
	3.0	6.2	31.2	35.3
	4.5	6.0	27.8	28.2
	6.0	4.0	19.4	16.8
	7.5	2.3	13.5	11.1
Yellow Shellac Microcapsules	1.5	5.3	35.1	19.6
	3.0	6.8	24.4	22.9
	4.5	2.8	13.3	7.8
	6.0	2.2	11.3	4.7
	7.5	4.1	17	9.7

**Table 5:** Significance analysis of the gloss of coatings with the different content of different shellac microcapsules based on the Beli wood surface.

Different Source	SS	d_f_	MS	F	*p*-value	F_crit_
Microcapsule Content	1063.35	4.00	265.84	8.64	0.01	3.84
Microcapsule Type	213.20	2.00	106.60	3.47	0.08	4.46
Error	246.01	8.00	30.75	-	-	-
Total	1522.56	14.00	-	-	-	-

**Table 6:** The hardness of the coating on Beli wood with the content of different shellac microcapsules in the coating.

Microcapsule Content (%)	Hardness		
	Transparent Shellac Microcapsules	Purple Shellac Microcapsules	Yellow Shellac Microcapsules
0	B	B	B
1.5	HB	B	HB
3.0	H	HB	H
4.5	H	H	2H
6.0	2H	H	2H
7.5	2H	2H	3H

**Table 7:** The adhesion of the coating on Beli wood with the content of different shellac core microcapsules in the coating.

Microcapsule Content (%)	Adhesion (Grade)		
	Transparent Shellac Microcapsules	Purple Shellac Microcapsules	Yellow Shellac Microcapsules
0	1	1	1
1.5	1	1	1
3.0	2	1	2
4.5	2	2	2
6.0	3	2	4
7.5	3	4	4

**Table 8:** The impact resistance of the coating on Beli wood with the content of different shellac core microcapsules in the coating.

Microcapsule Content (%)	Impact Resistance (kg·cm)		
	Transparent Shellac Microcapsules	Purple Shellac Microcapsules	Yellow Shellac Microcapsules
0	7	7	7
1.5	7	7	8
3.0	8	7	8
4.5	6	6	7
6.0	6	5	6
7.5	5	4	4

**Table 9:** The ΔE* of coating on Beli wood with the content of different shellac microcapsules in the coating after the cold liquid resistance test.

Microcapsule Type	Microcapsule Content (%)	ΔE* after Cold Liquid Resistance			
		Soy Sauce	Toilet Water	Iodine	75% Medical Ethanol
Transparent Shellac Microcapsules	0	6.68	9.65	12.01	17.24
	1.5	3.02	2.50	6.81	2.89
	3.0	3.05	14.58	11.15	9.33
	4.5	4.71	7.16	2.74	2.43
	6.0	8.96	8.25	8.01	10.01
	7.5	2.30	6.44	4.28	4.16
Purple Shellac Microcapsules	1.5	14.08	10.67	14.11	14.44
	3.0	6.05	3.71	6.49	4.07
	4.5	6.37	5.76	7.50	6.57
	6.0	11.46	9.20	9.68	10.83
	7.5	9.36	14.77	4.40	7.90
Yellow Shellac Microcapsules	1.5	2.20	3.23	2.45	4.30
	3.0	8.58	7.45	7.87	9.66
	4.5	9.53	10.27	6.06	11.20
	6.0	8.67	4.81	9.25	7.05
	7.5	14.94	10.86	15.10	12.96

**Table 10:** The cold liquid resistance of coating on Beli wood with the content of different shellac microcapsules in the coating.

Microcapsule Type	Microcapsule Content (%)	Cold Liquid Resistance (Grade)			
		Soy Sauce	Toilet Water	Iodine	75% Medical Ethanol
Transparent Shellac Microcapsules	0	1	1	1	1
	1.5	1	1	2	1
	3.0	3	1	2	1
	4.5	2	1	2	1
	6.0	1	1	1	1
	7.5	2	1	1	1
Purple Shellac Microcapsules	1.5	1	1	1	1
	3.0	2	1	2	1
	4.5	1	2	2	1
	6.0	1	1	2	1
	7.5	2	1	1	1
Yellow Shellac Microcapsules	1.5	2	1	2	2
	3.0	1	1	2	1
	4.5	2	1	1	1
	6.0	1	2	2	1
	7.5	1	1	2	1

**Table 11:** The mechanical qualities of the coating on Beli wood before and after aging.

Microcapsule Type	Microcapsule Content (%)	Aging Time (h)	Hardness	Adhesion (Grade)	Impact Resistance (kg·cm)	Roughness (μm)
Transparent Shellac Microcapsules	0	0	B	1	7	0.58
		36	2H	1	4	0.76
	3.0	0	H	2	8	1.31
		36	2H	3	6	0.98
	6.0	0	2H	3	6	1.52
		36	3H	4	5	1.67
Purple Shellac Microcapsules	3.0	0	HB	1	7	1.19
		36	H	2	5	0.85
	6.0	0	H	2	5	1.51
		36	2H	2	4	1.63
Yellow Shellac Microcapsules	3.0	0	H	2	8	1.41
		36	2H	3	6	1.50
	6.0	0	2H	4	6	1.79
		36	3H	4	5	2.31

## Data Availability

Not applicable.
